# Overcoming Microalgae Harvesting Barrier by Activated Algae Granules

**DOI:** 10.1038/s41598-017-05027-3

**Published:** 2017-07-05

**Authors:** Olga Tiron, Costel Bumbac, Elena Manea, Mihai Stefanescu, Mihai Nita Lazar

**Affiliations:** 1 Department of Environmental Technologies and Technological Transfer, National Research and Development Institute for Industrial Ecology - ECOIND, 71–73 Drumul Podu Dambovitei, 060652 Bucharest, Romania; 2 Department of Pollution Control, National Research and Development Institute for Industrial Ecology - ECOIND, 71–73 Drumul Podu Dambovitei, 060652 Bucharest, Romania

## Abstract

The economic factor of the microalgae harvesting step acts as a barrier to scaling up microalgae-based technology designed for wastewater treatment. In view of that, this study presents an alternative microalgae-bacteria system, which is proposed for eliminating the economic obstacle. Instead of the microalgae-bacteria (activated algae) flocs, the study aimed to develop activated algae granules comprising the microalgae *Chlorella* sp. as a target species. The presence of the filamentous microalgae (*Phormidium* sp.) was necessary for the occurrence of the granulation processes. A progressive decrease in frequency of the free *Chlorella* sp. cells was achieved once with the development of the activated algae granules as a result of the target microalgae being captured in the dense and tangled network of filaments. The mature activated algae granules ranged between 600 and 2,000 µm, and were characterized by a compact structure and significant settling ability (21.6 ± 0.9 m/h). In relation to the main aim of this study, a microalgae recovery efficiency of higher than 99% was achieved only by fast sedimentation of the granules; this performance highlighted the viability of the granular activated algae system for sustaining a microalgae harvesting procedure with neither cost nor energy inputs.

## Introduction

**Figure 1:**
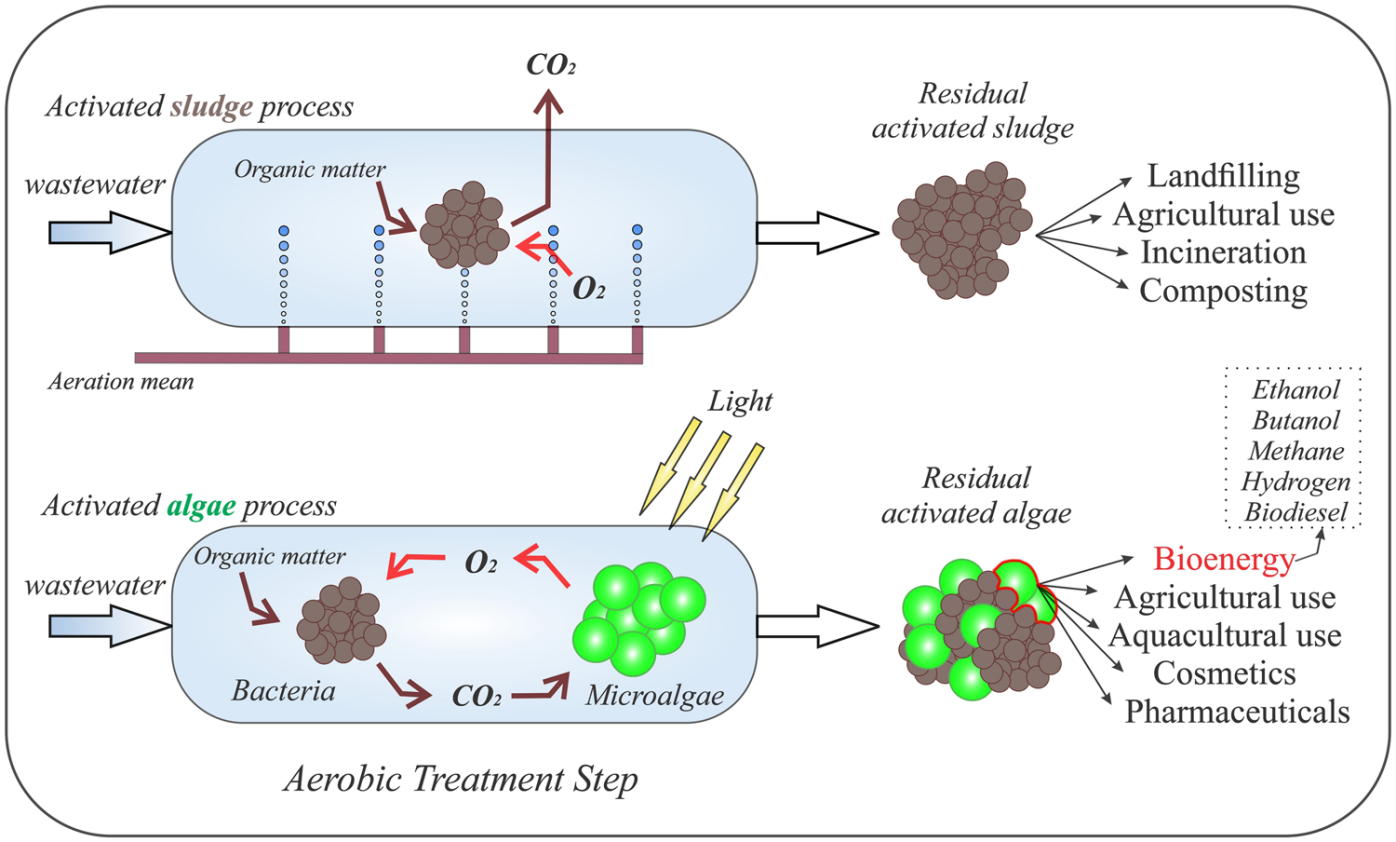
Microalgae-based technology for aerobic wastewater treatment. Economic and ecologic advantages that could be obtained by implementing activated algae-based technology in comparison with the conventional activated sludge process. Aerobic processes could occur in the activated algae system without mechanical aerations means as the oxygen is generated through photosynthesis processes, released CO_2_ during wastewater treatment is used in photosynthesis by microalgae, and residual microalgae biomass could be used in a wide range of applications. In comparison, aerobic wastewater treatment using activated sludge requires a significant amount of energy for oxygen generation, causes the release of the greenhouse gas such as CO_2_, and capitalization of the residual activated sludge is limited by legislative norms^41^.

Integrating microalgae biomass production with the wastewater treatment step is a promising approach for the implementation of a sustainable wastewater treatment technology that considers economical, ecological, and social impacts. This statement is based on the fact that microalgae-bacteria processes could sustain a greenhouse gas mitigation strategy^1^ and the elimination of the aeration costs with simultaneous production of a valuable residual biomass that could be used for the production of bioenergy and high-value chemicals^2, 3^ (Fig. 1). Moreover, the production of microalgae biomass during wastewater treatment is considered as a condition for sustaining the economic viability of algae-based biofuel production^4^, microalgae biomass being classified as a third generation renewable resource for bioenergy production^5^. Microalgae-bacteria flocs developed during wastewater treatment are called ‘activated algae’ flocs, the term being used for the first time several decades ago during experimental research performed by McGriff and McKinney^6^.

However, wastewater treatment technology based on activated algae processes faces several drawbacks. Microalgae harvesting is one of the major constraints in microalgae biotechnology development at an industrial scale^7, 8^ due to the substantial costs and energy involved in this step. Frequently applied harvesting techniques can account for between 20 and 30% of total microalgae cultivation costs^9^. Moreover, the economic impact of the microalgae harvesting step compromises the development of a microalgae-based biofuel production industry^10^, with up to 50% of the total costs for biofuel production being attributed to the harvesting step^11^. This economic inefficiency is caused by the low settling velocity of the microalgae (lower than 0.0036 m/h) and their density, which is similar to that of water^12^, with the microalgae species commonly used in wastewater treatment processes and the bioenergy production sector usually having a cell size of less than 30 µm^13^.

No universal method was delineated for the microalgae harvesting step^14^, and several physical, chemical, and biological techniques have been proposed to date. Processes such as centrifugation, chemical coagulation-flocculation, filtration, gravity sedimentation, and flotation represent frequently applied harvesting techniques^15^. Centrifugation is known as the most time-efficient method, and also as the most energy-intensive when it is applied as a primary step for microalgae harvesting; an energy consumption of higher than 3,000 kWh/t microalgae has been reported^16^. Compared with centrifugation, chemical flocculation requires less energy^10^. However, the method presents several important drawbacks, such as end-product contamination with metal salts, high costs for flocculant acquisition (especially at an industrial scale), as well as the sensitivity of the procedure for the following factors: pH, the morphology of the microalgal cells, the stage of the microalgae life cycle, and flocculant type^17, 18^. Gravity sedimentation is applied for activated sludge recovery, being the most cost-suitable harvesting method. However, in the case of the activated algae process, it is best applied to algae species larger than 70 µm^5^, otherwise it becomes a very time-consuming method (several days), which compromises cell integrity. In comparison with sedimentation, the flotation technique is considered to be more effective^19^.

Bio-flocculation represents an environmentally friendly harvesting technique comprising microalgae cells aggregation with different microorganisms, such as bacteria, autoflocculating microalgae and filamentous fungi. Therefore, use of microalgae species in wastewater treatment processes leads to an increase in microalgae recovery efficiency by developing the microalgae-bacteria flocs. For instance, the microalgae-bacteria flocs used by de Godos *et al*.^20^ for wastewater treatment exhibited a settling rate of 0.28–0.42 m/h.

Taking into consideration the particularities of the discussed harvesting techniques, as well as the emerging studies in the microalgal biotechnology field, it seems that none of the proposed harvesting techniques are economically and/or ecologically viable at an industrial scale; therefore, the development of a cost and energy efficient, effective, and environmentally friendly harvesting technique continues to be one of the most discussed strategies in microalgae-based technology.

This study focused on developing and describing an alternatively activated algae system, comprising granular entities, proposed as a solution for sustaining a cost and energy efficient microalgae harvesting step. The assessment of the activated algae granules’ viability for microalgae biomass recovery was conducted by presenting the main granules features, such as morphology, biological structure and settleability, and by analyzing the microalgae recovery efficiency that could be obtained after granules sedimentation.

## Results and Discussion

### Evolution of the activated algae flocs size *vs*. granules

*Chlorella* sp. represents one of the smallest taxa from the microalgae category. In the hereby conducted experiments, the size of the *Chlorella* sp. cells ranged between 1.3 and 7.6 µm, with mean cell size being 3.51 ± 0.18 µm.

**Figure 2:**
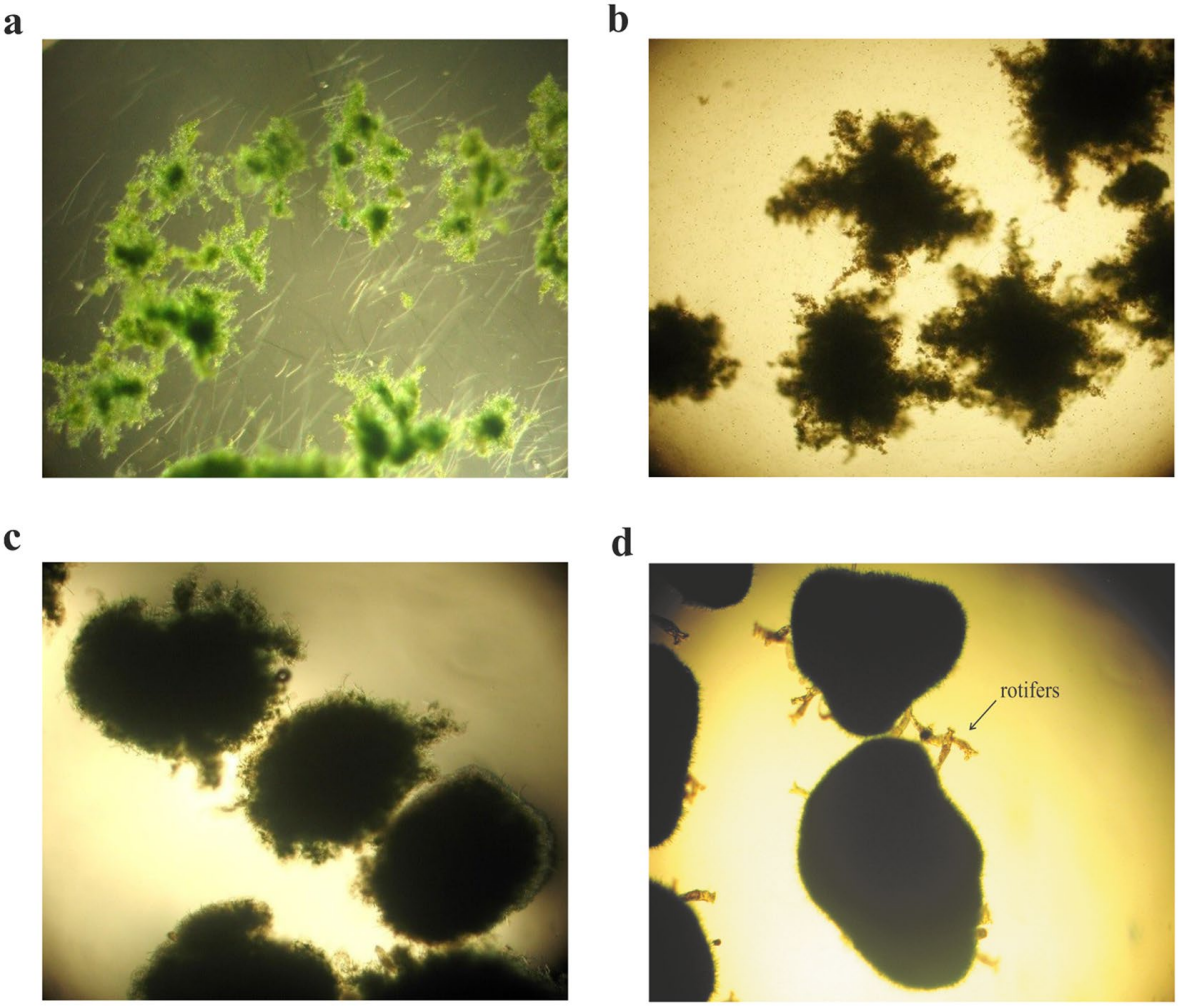
Transition of the activated algae system from floc-type to granular structure. Light microscopy images (40x magnification) of the activated algae aggregates during granulation step with emphasis on: (**a**) activated algae flocs (inoculum), (**b**) developed activated algae aggregates after 59 batches, and (**c**) 122 batches, and (**d**) mature activated algae granules (batch 153).

**Figure 3:**
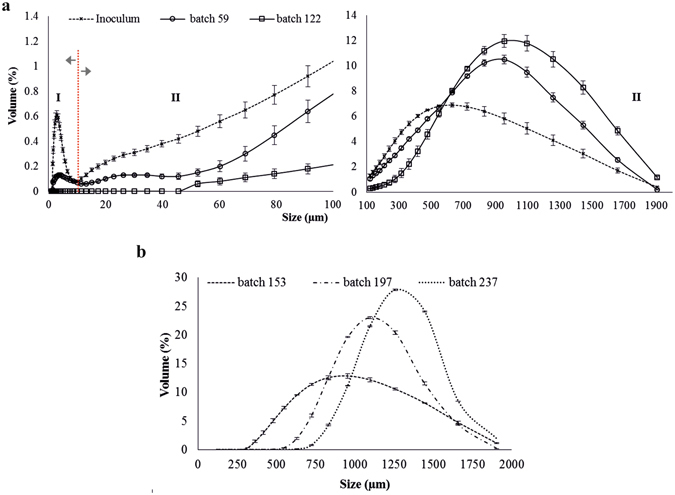
Size evolution of the activated algae granules. (**a**) Volume distribution of the target microalgae cells size (*section I*) and activated algae flocs size (*section II*) during the granulation procedure (the first 122 batches). (**b**) Dynamics of the volume distribution curve of the activated algae granules size recorded for the following culturing batches: 153, 197, and 237.

The activated algae system used as an inoculum in the granulation process was represented by irregular, mainly open flocs, with a high frequency of the filamentous microalgae and target microalgae (Fig. 2a). A high abundance of filaments and free *Chlorella* sp. cells was also recorded in the liquor. The volume distribution of the activated algae flocs size is illustrated in Fig. 3a. The distribution curve presented two sections: *section I*, which represented the volume distribution of the free target microalgae cells size, and *section II*, which represented the volume distribution of the activated algae flocs size. According to the recorded results, only a small percentage (1.26 ± 0.06%) of the total volume was attributed to the flocs under 25 µm, with the highest proportion (78.8 ± 1.21%) being represented by large flocs (>250 µm), the remaining percentage of 19.9 ± 1.12% comprising medium-sized flocs (25–250 µm). The mean size of the activated algae flocs was 598.24 ± 22.71 µm.

With the increase in number of the culturing batches during the granulation process, a decrease in distribution of the free target microalgae was noticed. As a result, after 59 culturing batches, the volume of the *Chlorella* sp. cells outside flocs was 74.6 ± 2.3% lower compared to the first batch. In spite of this sharply decreasing tendency, the presence – with a high frequency – of free target cells in liquor was still noticed. Compared with the first batch, after 59 batches, the volume of the small and medium-sized activated algae flocs decreased by about 52.3 ± 1.6 and 36.5 ± 0.63%, respectively. As a result, the volume of the large-size flocs increased by 22.2 ± 1.16% and the mean size of the activated algae flocs increased to 730.62 ± 11.28 µm. The microscopy investigations underlined an evolution of the flocs from an irregular shape to a round shape. Floc structure remained open, being governed by the filaments’ development (Fig. 2b).

After 122 batches, the round shape of the activated algae aggregates was clearly defined (Fig. 2c) and complete removal of particles smaller than 10 µm was obtained (Fig. 3a). These results showed that target microalgae cells were efficiently entrapped into activated algae aggregates whose mean size increased to 968.3 ± 19.7 µm. Moreover, no presence of the small flocs was detected, the activated algae system being mainly represented by large flocs (96.6 ± 0.26%), with the volume of medium-sized flocs being 3.41 ± 0.25%. At this stage, development of compact flocs was emphasized.

At the applied operational conditions, the granulation of the activated algae flocs was considered accomplished after 153 culturing batches. At this stage, granules size was higher than 300 µm, with almost 65.9 ± 2.3% of them ranging between 720 and 1,450 µm. The mean size of the granules was 1,034.8 ± 1.9 µm. Developed activated algae granules were characterized by a compact structure (Fig. 2d). During this stage, a high frequency of rotifer population was also noticed.

**Figure 4:**
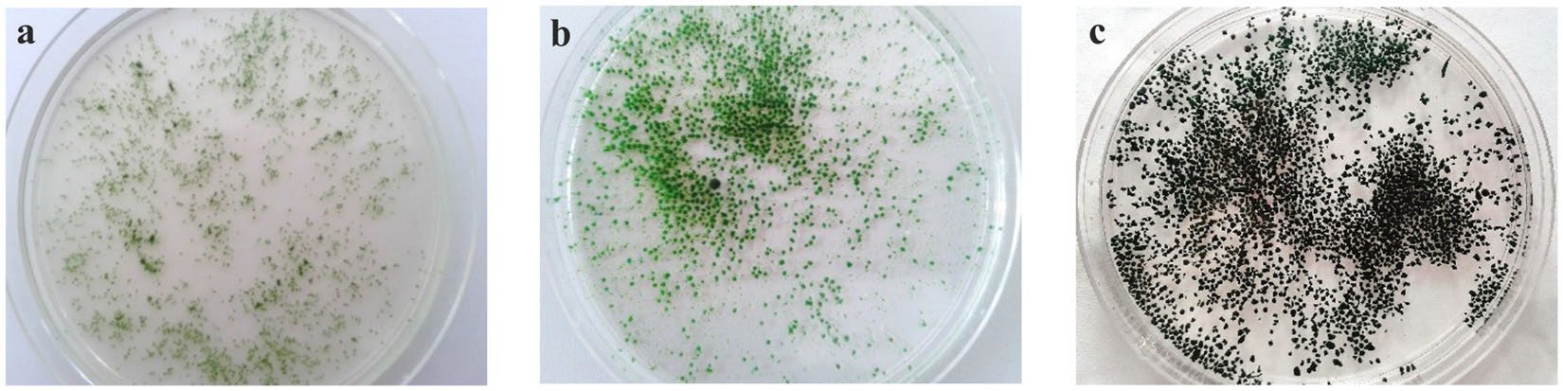
The time development of the granular activated algae system. Activated algae system during granulation process: (**a**) batch 59, (**b**) batch 122, and (**c**) obtained activated algae granules after 197 batches.

Further culturing of the activated algae granules contributed to the increase of the granules’ mean size to 1,176.1 ± 5.4 (batch 197) and 1,343.7 ± 1.8 µm (batch 237; Fig. 3b). Moreover, light microscopy images showed an increase of granule compactness level, property also underlined by the dark-green colour of the granules (Fig. 4c) compared to the light green observed during the granulation process (Fig. 4a,b). The dry weight of the granules, reached after 197 batches, was found to be 75.6 ± 17.6 µg/granule; at the end of the experimental part of the study, granule weight increased to 155.7 ± 20.9 µg/granule.

It is worth pointing out that after 237 batches, the volume of the granules with a diameter between 720 and 1,450 µm increased to 90.4 ± 1.13%. Moreover, during the entire culturing period (assigned to the granulation process), the volume of the granules larger than 1,450 µm was kept lower than 11%, with less than 2% reaching 1,900 µm. Taking into consideration the results stated above, it could be concluded that the optimum size of the granules ranged between 700 and 1,500 µm. An increase in granule size to above 1,500 µm could act as a limiting factor for granule development, possibly due to the decrease of nutrients and light diffusion caused by the highly compact structure. However, the adaptation processes could strongly influence the evolution of the volume distribution curve of the granule size. Once with granules formation, due to light and nutrients diffusion gradients, different growth conditions (aerobic, anoxic, anaerobic) could be achieved causing the organization of aggregates into multilayer structures, each layer providing conditions for the development of the specific microorganisms populations. As a result, the development of granules above 2,000 µm could be expected, with further research being needed in this case.

### Properties of the activated algae granules

**Figure 5:**
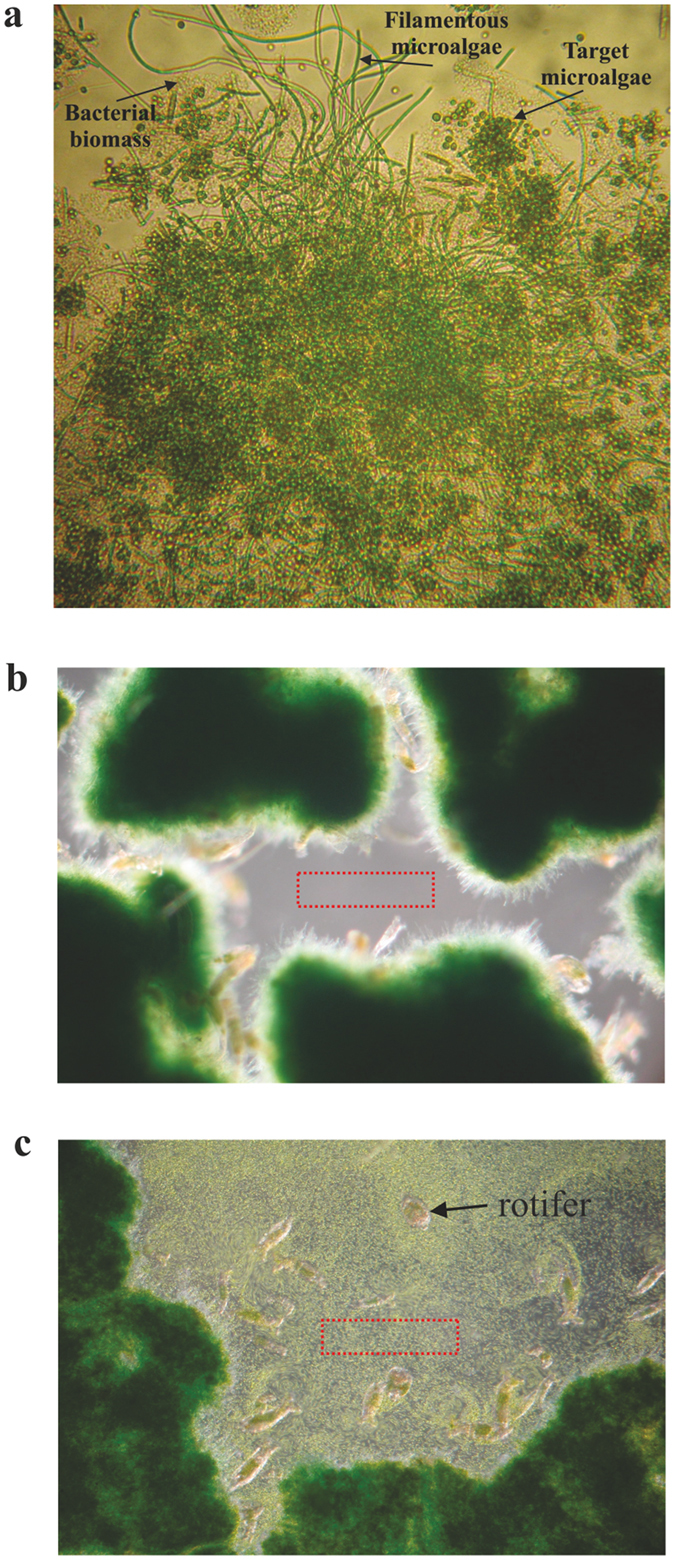
Biological structure of the activated algae granule with the performance achieved in target microalgae capturing inside granular structure. Light microscopy images of a crushed activated algae granule with emphasis on (**a**) target microalgae *Chlorella* sp., filamentous microalgae, and bacterial biomass (200x magnification), and (**b**) of the activated algae granules (40x magnification), (**c**) a rich population of the target microalgae *Chlorella* sp. being released after crushing of mature granules. Red dotted rectangle highlights the difference between the liquor’s charge in target microalgae cells before and after granules crushing.

**Figure 6:**
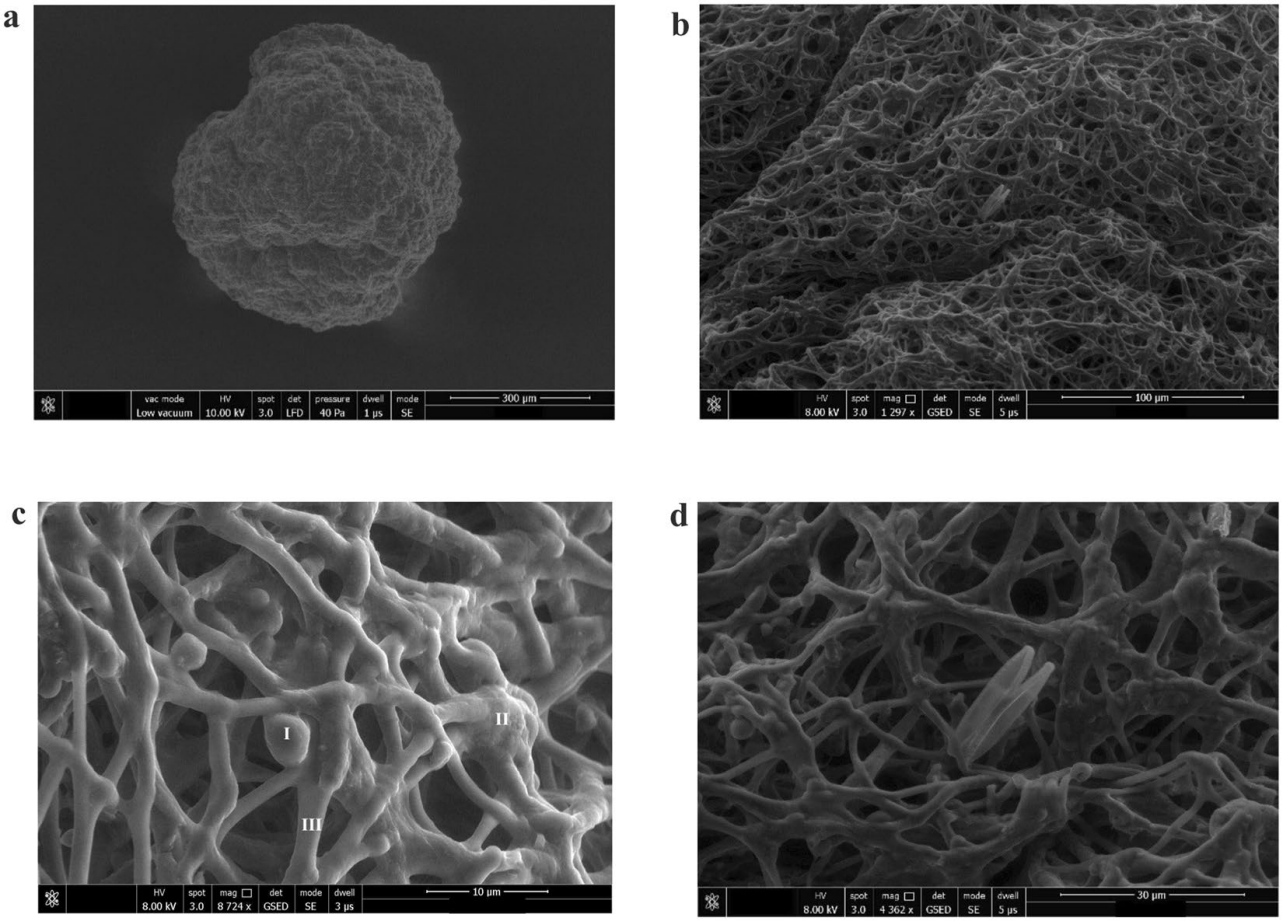
SEM images of the activated algae granule. (**a**) Activated algae granule with emphasis on (**b**) granule structure, and (**c**) inside populations: target microalgae *Chlorella* sp. (I), bacterial biomass (II), filamentous microalgae (III), and (**d**) *Nitzchia palea* diatoms.

Light microscopy images of the crushed activated algae granules showed the presence of bacteria – target microalgae *Chlorella* sp., and filamentous microalgae *Phormidium* sp. – populations inside granules (Fig. 5a). According to the SEM images, it was concluded that the granular structure was developed through both non-flocculative and bioflocculative processes. The non-flocculative process comprised the microalgal filaments interwoven in a tangled, dense, globular network (Fig. 6a,b), the set-up stirring conditions being one of the key factors for the occurrence of this process. As a result, during the activated algae granules’ development, the free target microalgae cells were captured in a dense network of filaments, with the filamentous microalgae acting as an efficient ‘biological filter’. The performance achieved in this case was emphasized not only by the volume distribution curve of the activated algae aggregates size (as represented in Fig. 3a), but also by the light microscopy investigations. For instance, after granule crushing, the microscopy images revealed the release of a rich population of the target microalgae cells from the granular structure (Fig. 5b,c).

The bioflocculation process consisted of the aggregation of microalgae and bacteria biomass by released biopolymers. The occurrence of this process inside granules is shown in Fig. 6c. It was observed that the filaments acted as a biological support for both the microalgae and bacteria cells, promoting the development of these species inside granules. Therefore, besides the ‘biological filter’ role, the filamentous microalgae had a similar role to that of the filamentous bacteria present in conventional activated sludge flocs, where these species represent flocs’ ‘backbone’^21^. Moreover, the filamentous microalgae *Phormidium* sp. have the ability to self-aggregate^22^ and present a gelatinous matrix^23^, properties that could also influence the efficiency of the target microalgae entrapment and maintenance inside the granular structure. In addition to the target microalgae, the presence of *Nitzschia palea* diatoms was also noticed inside the granules (Fig. 6d). Thus, the developed activated algae granules could be considered for harvesting larger-size species than *Chlorella* sp., according to the microscopy investigations, with the presented diatoms having a cell size greater than 4 µm wide and 20 µm long.

Developing the activated algae flocs and activated algae granules in real dairy wastewater promoted the development of a viable biomass, with potential application in wastewater treatment processes. The viability of the developed activated algae granules in wastewater treatment processes have been proven in our previous research^24^ where the symbiotic relationship developed between bacteria and microalgae inside the granular structure contributed to: (1) almost complete removal of organic matter, using only the oxygen provided through photosynthesis processes; (2) the complete removal of ammonium, with the performance promoted through cell assimilation and nitrification processes; and (3) the occurrence of the denitrification processes sustained by the variation of oxygen saturation during the conducted treatment batches and possibly by the dense granular structure. In addition to the role of the *Phormidium* sp. taxa in sustaining the granular structure, the cyanobacterial populations were also involved in wastewater treatment processes along with the target microalgae cells, with both populations representing oxygen-producing microorganisms. The co-existence between filamentous cyanobacteria *Phormidium* sp. and other microalgae could have been achieved due to the fact that the *Phormidium* genus is characterized by a high tolerance for microalgae diversity, as shown by Pouliot *et al*.^25^. Thus, *Phormidium* sp. could be considered for sustaining in each granule the development of a stable trophic network. Moreover, these properties could play an important role in the analysis of biotechnology viability beyond laboratory scale, where the microalgae community could undergo severe taxonomic changes.

However, more research is still required for decreasing the duration of granulation process (from suspended biomass to granules). Possible industrial scale start-up of a wastewater treatment plant based on activated algae granules could be performed either from suspended biomass followed by granulation, either by inoculating with previously developed granules (in pilot scale or in another wastewater treatment plant).

Based on the SEM investigations, it could be assumed that a ‘porous’ granular structure could promote a more efficient diffusion of the organic matter, nutrients and oxygen inside granules compared to granular activated sludge. This property could have a considerable influence on granules’ speciation and on the efficiency of their use in wastewater treatment processes, with further investigations being required in this case.

### Granules settleability

**Figure 7:**
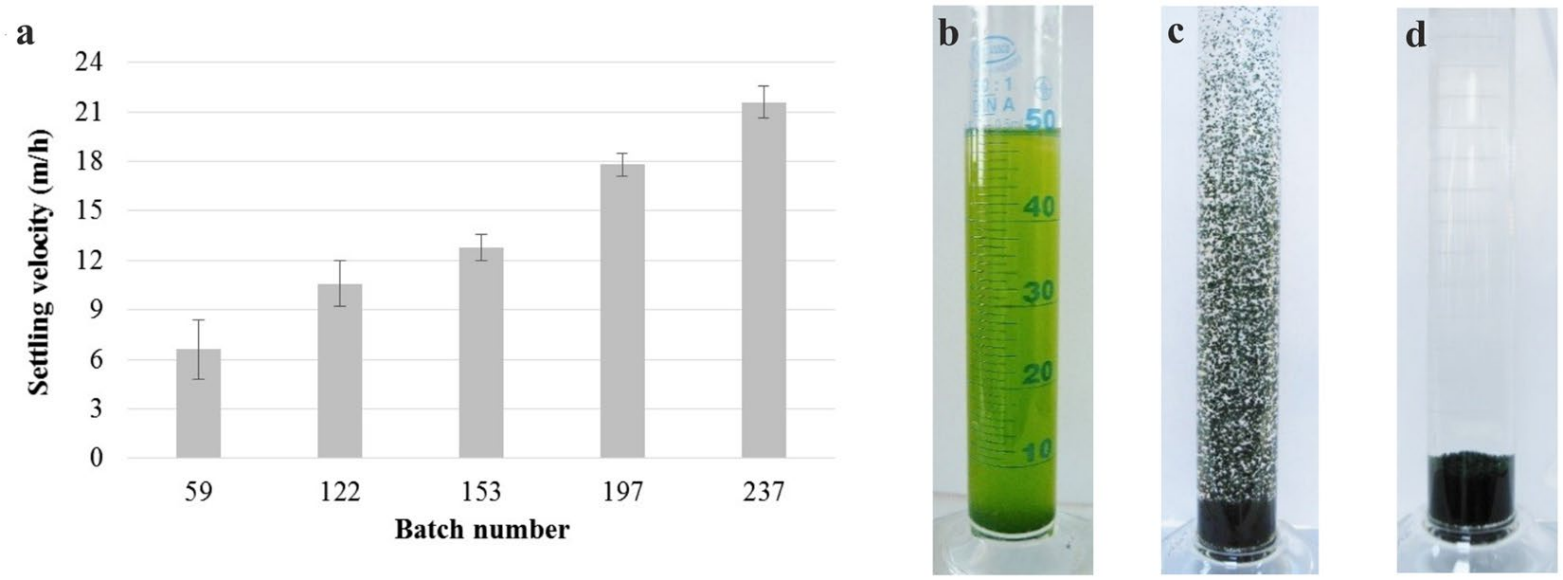
The influence of activated algae granules development on biomass settling velocity. (**a**) Settling rate of the activated algae biomass recorded during the granulation procedure in respect to the investigated batch number. (**b**) Sedimentation of the activated algae flocs (after 1 h), the free target microalgae cells (*Chlorella* sp.) remaining suspended due to their small cell size, and mature activated algae granules: (**c**) in the first seconds of the settling phase and (**d**) after 2 min of settling.

The settling rate of the target microalgae cells *Chlorella* sp. was found to be lower than 0.54 × 10^−2^ m/h. In comparison with the target microalgae, the settling rate of the filamentous cyanobacteria *Phormidium* sp. could reach 12 m/h^25^, mostly due to their self-aggregation ability, morphology and larger size (2–10 µm wide, 100–200 µm long)^26^. The settling rate of the activated algae biomass progressively increased with batch number from 6.6 ± 1.8 m/h (batch 59) to 21.6 ± 0.9 m/h (batch 237; Fig. 7a). The performance achieved in this case, by switching from activated algae flocs to activated algae granules, is illustrated in Fig. 7b–d.

Granados *et al*.^12^ stated that an efficient microalgae settleability could be achieved at a settling rate higher than 0.36 m/h. Therefore, this condition could be accomplished using activated algae granules, with no chemicals or energy inputs required during harvesting step. Moreover, the settling velocity of the mature activated algae granules was higher than that reported in the case of the conventional activated sludge flocs (7.2–10.8 m/h)^27^ and was comparable to that recorded in the case of the aerobic granular sludge (18–32.4 m/h)^28^.

### Microalgae recovery efficiency

Due to the small cell size, harvesting *Chlorella* sp. biomass is an expensive procedure. Even the centrifugation method is insufficient for harvesting these microalgae species^29^, with application of the combined methods being necessary to increase recovery efficiency. However, the use of two or more harvesting techniques rarely contributes to a microalgae recovery efficiency higher than 95%.

In this study, the residual chlorophyll *a* concentration found in effluents, after activated algae granules settling, ranged between 2.65 ± 0.41 and 54.13 ± 6.9 µg/L, with the mean result being 21.8 ± 2.3 µg/L. As a result, the microalgae recovery efficiency varied between 99.24 ± 0.08 and 99.93 ± 0.014%, with a mean value of 99.63 ± 0.029%. The results obtained suggest that the granulation of the activated algae flocs using filamentous microalgae (*Phormidium* sp.) represents a viable solution for almost complete recovery of the microalgae biomass (and implicitly of the target *Chlorella* sp. cells) using gravity sedimentation as a single harvesting technique. The development of the dense activated algae granules could be considered a promising alternative solution, not only for saving both costs and energy in the harvesting step, but also in the microalgae dewatering step, which is another barrier for the development of microalgae-based technology^30^, with further research being needed to sustain this statement.

As reported above, the activated algae biocenosis included rotifer populations. The presence of these species in liquor could influence microalgae recovery performance by capturing the remaining free target microalgae cells, as rotifers are well known for being grazers of these species^3^.

In practice, the optimum harvesting method is chosen based on several factors, such as microalgae species, cell density and culturing conditions^7, 8^. Using activated algae granules, the previously mentioned conditions could be overlooked. Moreover, taking into consideration the presented granules characteristics, it could be stated that the granulation procedure could be applied in the case of other *Chlorella*-like microalgae with a similar cell size characterized by poor settleability.

Almost complete microalgae recovery was also reported by Zhou *et al*.^31^ in the case of *Chlorella vulgaris* biomass. The microalgae harvesting was performed by fungi-algae pelletization. The principle of the hereby applied granulation procedure was similar to that reported in the case of fungal-assisted algae pelletization, with the microalgae recovery efficiency being reliant on the spores’ coagulation and hyphae intertwining processes^11^. However, the fungal-based pelletization procedure is highly sensitive to pH variation, with the optimal pH for fungi-algae pellets being found in acidic conditions, and pellet integrity being compromised at pH values higher than 7.5^31^. Acidic conditions (pH 3.5–5) obtained by the co-cultivation of the fungal biomass with microalgae (*Chlorella vulgaris*) was also reported by Xie *et al*.^32^. This particularity could be a major barrier for the integration of algae-fungal pellets into conventional wastewater treatment processes, with the efficiency of the activated sludge process decreasing at pH values lower than 6.5. Moreover, the optimal activity of several important bacteria (such as nitrifiers) occurred between 7.2 and 8.0 pH values^33^. Compared with fungi-algae pellets, the activated algae granules were developed at common pH values registered during conventional wastewater treatment processes (pH 6.5–8.5). Moreover, our previous investigations showed a high tolerance of granule structure at higher variations of pH (5–8.5)^24^. Such properties sustain the feasible character of the activated algae granules for further application in wastewater treatment processes. Further investigations must be taken into consideration for describing the response of the granular activated algae system at industrial scale conditions.

Considering potential industrial scale application for wastewater treatment, the harvesting process of activated algae granules can be performed during the settling phase (considering SBR operation) or in the secondary settler (for continuous flow conditions).

## Methods

### Materials

The granulation process was performed using conventional activated algae flocs as inoculum. Taking into consideration that this approach was followed in order to use the proposed method to develop activated algae granules for wastewater treatment, it was considered appropriate to use activated algae flocs, which comprise native conventional microalgae and bacteria species adapted to wastewater treatment conditions. As a result, microalgae and bacteria taxa were obtained by sampling a biofilm developed on the inner wall of a laboratory-scale sequencing batch reactor used for dairy wastewater treatment. In this study, conventional microalgae were considered to be any microalgae species smaller than 30 µm, which could be used in wastewater treatment processes, except filamentous microalgae.

The hereby discussed method of activated algae flocs granulation is a biological method that relies on the use of filamentous microalgae. Therefore, besides conventional microalgae and bacteria species, it was necessary to include filamentous microalgae in the biomass. The microscopic investigations performed on sampled biofilm showed the presence of all the above-mentioned populations (conventional microalgae, filamentous microalgae, and bacteria). Thus, in this study, it was not necessary to use an external source of filamentous microalgae. According to the microscopic investigations, the filamentous microalgae were represented by cyanobacteria populations *Phormidium* sp. The prevalent genus from the *Chlorophyte* category was *Chlorella* sp., single-cell species, which are frequently used in wastewater treatment processes, as well as for biomass production^34^. In view of the above findings, *Chlorella* sp. taxa were chosen as target microalgae.

### Experimental set-up

**Figure 8:**
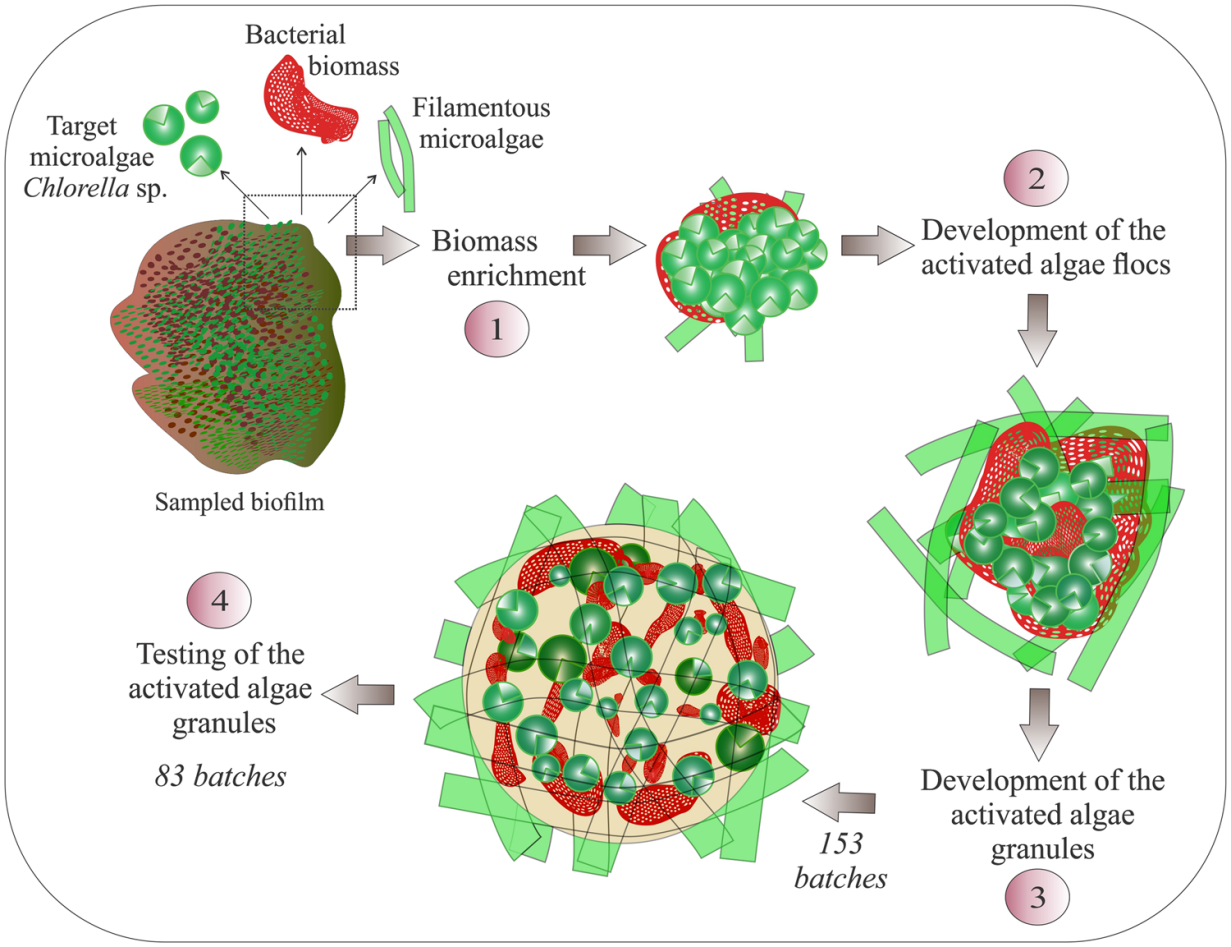
Schematic illustration of the experimental steps. Step 1 – cultivation of the microalgae – bacteria inoculum; step 2 – development of the conventional microalgae-bacteria system; step 3 – implementation of the activated algae granulation procedure; step 4 – validation of the obtained alternative activated algae system in downstream process (microalgae harvesting).

The experimental part of the study comprised four major steps, which are illustrated schematically in Fig. 8.

#### Step 1

The sampled biomass was enriched in mixed raw dairy wastewater – *Chlorella* broth media using Erlenmeyer flasks (250 mL), as described in our previous research^35^. This step was conducted to increase the biomass of the target microalgae while simultaneously maintaining the native bacterial populations. The biomass cultivation was carried out for about 3 months.

#### Step 2

The biomass obtained from the first step was used for the development of the conventional activated algae flocs. The process was carried out in a 4 L stirred tank photobioreactor (PBR) B*plus* (BIOSTAT^®^, Sartorius, Germany). The development of the activated algae flocs was carried out in sequential batch operation mode (SBR), with each culturing batch consisting of the following consecutively applied phases: (I) PBR feeding with 2 L of pretreated, unsterilized dairy wastewater (10 min), (II) reaction phase (83 h 40 min), liquor being stirred at a rate of 75 rpm, (III) settling phase (12 h), and (IV) effluent withdrawing (10 min) with the suspended biomass, with the settled biomass being used for the next batch. The photosynthetic light was provided by an exterior, cool-white fluorescent lamp (Model FQT8-55 E27, Lumen), with a light intensity of 235 µmol/m^2^/s at the outer wall of the vessel. During the reaction phase, the photoperiodicity was 15 h light: 9 h dark, with the light cycle beginning immediately after PBR feeding. During biomass enrichment, the liquor’s temperature ranged between 22 and 28 °C corresponding to light/dark cycles. Cultivation of the activated algae flocs was performed in the absence of mechanical aeration, with the oxygen supply being provided by microalgae through the photosynthesis processes. This step was conducted for about two months until activated algae flocs and a high frequency of filamentous microalgae species were obtained.

#### Step 3

Granulation of the activated algae flocs was performed in a 1.5 L PBR A*plus* (BIOSTAT^®^, Sartorius, Germany), in SBR operation mode, with employment of 153 culturing batches. The PBR was fed with pretreated dairy wastewater, as with the activated algae flocs development. The set-up operational conditions during granulation were detailed in our previous study^24^. Hydraulic retention time was decreased successively from 96 to 72, 48, and 24 h based on treatment performance. The liquor was stirred at a rate of 150 rpm and the settling phase was decreased to 1 h. The photosynthesis was sustained by an exterior, cool-white fluorescent lamp as it was described in the case of the activated algae flocs development (Step 2). Photoperiodicity was maintained at 15 h light: 9 h dark. The experiment was conducted for about 6 months.

#### Step 4

The activated algae granules obtained after 153 culturing batches were further cultured in SBR mode, in 1 L of 10-fold diluted dairy wastewater supplemented with NH_4_Cl (45 mg/L), MgSO_4_· 7H_2_O (100 mg/L), K_2_HPO_4_ (15 mg/L), and CaCl_2_· 2H_2_O (30 mg/L), to increase the concentration of NH_4_^+^, Mg^2+^, PO_4_^3−^, and Ca^2+^, respectively. As a result, the physicochemical parameters of the influent varied at the following intervals: pH (6.5–7.3), O_2_ (1–3%), chemical oxygen demand (COD; 127.4–195.6 mg O_2_/L), NH_4_^+^ (15.3–21.8 mg/L), NO_2_^−^ (<0.1 mg/L), NO_3_^−^ (<0.1 mg/L), PO_4_^3−^ (7.3–14.2 mg/L). The set-up operational conditions were as applied during activated algae granulation, except that the hydraulic retention time was maintained at 23 h 35 min, the liquor was stirred at a frequency of 120 rpm, and the time allocated for the granules’ sedimentation was 5 min. The PBR feeding and effluent withdrawing phases lasted 10 min each. In these conditions, 83 batches were conducted. During this step, the research mainly focused on the following issues: the determination of the microalgae recovery efficiency after the activated algae granules’ sedimentation (during the settling phase), the investigation of the dynamics of the volume distribution curve of the granules’ size, the microscopy examination of the mature activated algae granules, and the monitoring of the granules’ settleability and weight.

### Microalgae recovery efficiency

The microalgae recovery efficiency was determined based on chlorophyll *a* concentration^24^, according to the following equation:

1 $$R=[(Chl{a}_{I}-Chl{a}_{F})/Chl{a}_{I}]\cdot 100 \% ,$$

where *R* represents the microalgae recovery efficiency recorded after the activated algae granules’ sedimentation in the settling phase (5 min) (%), *Chla*_*I*_ is the chlorophyll *a* concentration of the granular activated algae biomass (mg/L), and *Chla*_*F*_ represents the residual chlorophyll *a* concentration from effluent withdrawn after granules settling (mg/L).

### Activated algae granule weight

The dry weight of the mature activated algae granule was calculated according to the method described in the previous study^35^ and by applying the following formula:

2 $$DW=[({F}_{90}-{F}_{0})\cdot {10}^{6}]/N,$$

where *DW* represents the dry weight of a granule (µg), *F*_*90*_ is the weight of the glass-microfibre filter (pore size 0.45 µm) with activated algae granules measured after drying at 90 °C for 24 h (g), *F*_*0*_ is the weight of the glass microfibre filter (g), and *N* represents the number of activated algae granules on the filter. The analysis was performed in triplicate.

### Particle size distribution and microscopy investigations

The volume distribution of the target microalgae cells, activated algae flocs and activated algae granules size was measured using analyzer Mastersizer 2000 (Malvern Instruments, Malvern, UK), based on the laser diffraction method, the refractive index being set at 1.060^36^. The biological samples were examined using the Optech B1 light microscope (Germany) and the scanning electron microscope (SEM) Quanta 250 FEG (FEI, Netherlands).

### Biomass settling velocity

250 mL of homogenous sample was transferred into a graduated cylinder (250 mL, 0.23 m height). The activated algae aggregates were allowed to settle and the biomass position in the cylinder was recorded during the settling time. The settling velocity of the activated algae aggregates was calculated as follows:

3 $$S=\frac{P}{{T}_{f}-{T}_{0}}$$

where *S* represents the settling velocity of the activated algae aggregates (m/h) and *P* is the height (m) crossed by biomass between initial time (*T*_*0*_) (h) and final time (*T*_*f*_) (h).

### Analytical methods

The pH and oxygen saturation values were determined by EasyFerm Plus K8 (200) electrode (Hamilton, Bonaduz, Switzerland) and OxyFerm FDA (225) sensor (Hamilton, Bonaduz, Switzerland), respectively. COD was analysed according to SR ISO 6060 standard^37^ based on the potassium dichromate method. The SR ISO 14911^38^ standard was used for measurement of NH_4_^+^concentration, while the SR EN ISO 10304/1^39^ standard was applied in the case of NO_2_^−^, NO_3_^−^, and PO_4_^3−^, analyses performed using the ion chromatography system ICS-3000 (Dionex, Sunnyvale, CA, USA). The chlorophyll *a* concentration was determined according to ISO standard SR 10260^40^ using UV-Vis spectrophotometer (Model DR/5000^TM^, Hach Lange, Düseldorf, Germany). The analyses was performed by using ethanol (≥96%) purchased from Chemical Company (Iasi, Romania).

All analyses were carried out on duplicate samples, the results being expressed as the means and standard errors.
